# Accumulation of Anthocyanin in the Aleurone of Barley Grains by Targeted Restoration of the *MYC2* Gene

**DOI:** 10.3390/ijms252312705

**Published:** 2024-11-26

**Authors:** Anastasiya A. Egorova, Tatyana E. Zykova, Christian W. Hertig, Iris Hoffie, Sergey V. Morozov, Elena I. Chernyak, Artem D. Rogachev, Anna M. Korotkova, Alexander V. Vikhorev, Gennady V. Vasiliev, Olesya Y. Shoeva, Jochen Kumlehn, Sophia V. Gerasimova, Elena K. Khlestkina

**Affiliations:** 1 Institute of Cytology and Genetics of the Siberian Branch of the Russian Academy of Sciences, 630090 Novosibirsk, Russia; egorova@bionet.nsc.ru (A.A.E.); t.zykova@g.nsu.ru (T.E.Z.); korotkova@bionet.nsc.ru (A.M.K.); olesya_ter@bionet.nsc.ru (O.Y.S.); khlest@bionet.nsc.ru (E.K.K.); 2 Faculty of Natural Sciences, Novosibirsk State University, 630090 Novosibirsk, Russia; moroz@nioch.nsc.ru (S.V.M.); vikhorev@bionet.nsc.ru (A.V.V.); 3 Leibniz Institute of Plant Genetics and Crop Plant Research (IPK), 06466 Gatersleben, Germany; hertig@ipk-gatersleben.de (C.W.H.); hoffiei@ipk-gatersleben.de (I.H.); kumlehn@ipk-gatersleben.de (J.K.); 4 N.I. Vavilov All-Russian Research Institute of Plant Genetic Resources, 190000 Saint Petersburg, Russia; 5 N.N. Vorozhtsov Novosibirsk Institute of Organic Chemistry, Siberian Branch of the Russian Academy of Sciences, 630090 Novosibirsk, Russia; chernyak@nioch.nsc.ru (E.I.C.); artrogachev@yandex.ru (A.D.R.)

**Keywords:** genome editing, Cas9/gRNA, pigmented barley, nutritional value, flavonoid biosynthesis, grain color

## Abstract

Blue barley grain pigmentation results from anthocyanin accumulation in the aleurone layer. Anthocyanins are known for their beneficial effects on human health. The gene encoding the MYELOCYTOMATOSIS 2 (MYC2) transcription factor is potentially responsible for the blue coloration of the aleurone. In non-pigmented barley, a single nucleotide insertion in this gene causes a frameshift mutation with a premature stop codon. It was hypothesized that restoring the *MYC2* reading frame could activate anthocyanin accumulation in the aleurone. Using a targeted mutagenesis approach in the present study, the reading frame of *MYC2* was restored in the non-pigmented cultivar Golden Promise. Genetic constructs harboring *cas9* and *gRNA* expression units were developed, pre-validated in protoplasts, and then functional *MYC2* alleles were generated at the plant level via *Agrobacterium*-mediated transformation. Anthocyanin accumulation in the aleurone layer of grains from these mutants was confirmed through microscopy and chemical analysis. The expression of anthocyanin biosynthesis genes was analyzed, revealing that the restoration of MYC2 led to increased transcript levels of *F3H* and *ANS* genes. These results confirm the critical role of the MYC2 transcription factor in the blue aleurone trait and provide a biotechnological solution for enriching barley grain with anthocyanins.

## 1. Introduction

The barley (*Hordeum vulgare* L.) grain contains a variety of bioactive compounds that contribute to its classification as a functional food ingredient [1,2,3,4,5]. In particular, phenolic compounds, especially those belonging to the flavonoid and anthocyanin groups, have demonstrated notable antioxidant activity in both in vitro and in vivo studies [6,7]. In barley plants, anthocyanins, which are secondary metabolites, are synthesized and accumulated in the grain husks, leaf sheaths, stems, and other tissues. This causes the emergence of various colors depending on the predominant pigment. In plants, anthocyanins regulate growth and development and provide protection against environmental impacts [8].

Blue color of barley grain is determined by the spectrum of anthocyanin pigments synthesized in aleurone cells under control of the five complementary *Blx* (*Blue aleurone xenia*) loci. These loci were mapped on chromosomes 4H (*Blx1*, *Blx3*, *Blx4*) and 7H (*Blx2*, *Blx5*) [9,10]. So far, molecular functions have been suggested for four of them. The MbHF35 cluster is located on chromosome 4H and has been reported to be responsible for the blue aleurone trait [11,12]. Two genes encoding the transcription factors MYELOCYTOMATOSIS 2 (MYC2) and R2R3-MYB (MYB4H) and a gene encoding the enzyme FLAVONOID 3′,5′-HYDROXYLASE (F3′5′H) are parts of this cluster. Jia et al. [11] suggested that they are candidates for *Blx1*, but according to Strygina et al. [12], *MYC2* is a candidate gene for *Blx1* or *Blx3*, while *F3′5′H* has been shown to be *Blx4*. *GST* (Glutathione S-transferase) is located on chromosome 7HL and has been suggested as a candidate for the *Blx2* locus [13]. Previous studies have demonstrated the tissue-specific co-expression of the *MYC2* transcription factor gene and genes encoding enzymes of the anthocyanin biosynthesis pathway [11,12]. Additionally, it was shown that a frameshift single nucleotide insertion in the *MYC2* gene is highly correlated with an uncolored grain phenotype, whereas the functional allele of this gene is associated with blue aleurone color [12]. Strong correlation of *MYC2* expression with anthocyanin biosynthesis genes was found in Tibetan hull-less barley [14]. In support of the role of the *MYC2* gene in blue color trait formation, the orthologues of this gene have been shown to regulate anthocyanin synthesis in the aleurone of other cereals. *TsMYC2* is identified as a candidate gene that controls the blue aleurone layer traits in triticale. The relative transcript level of *TsMYC2* in the blue aleurone line was found to be 42.71 times higher than in the white aleurone line. Through transient expression in white wheat coleoptile cells, *TsMYC2* was observed to induce anthocyanin biosynthesis [15]. Another ortholog, *ThMYC4E* originating from *Thinopyrum ponticum* was described as a candidate gene for the blue and purple grain traits in wheat genotypes with blue grain and *Th. ponticum* introgressions [16]. Overexpression of the *ThMYC4E* gene leads to elevated levels of purple anthocyanin pigments in the grains, leaves, and glumes of common wheat [17]. 

Genetic engineering is employed for the improvement of crop plants for a variety of purposes, including enrichment of plant raw materials with dietary valuable compounds. Examples of successful genome editing to enhance dietary value include increasing the content of carotenoids in tomatoes [18,19] and bananas [20], iron and zinc in wheat [21], improving sorghum digestibility [22], modifying starch and acids properties in rice [23,24,25,26] and corn [27], or even removing toxic components in potatoes [28,29] and oil seed crops [30]. It was shown that anthocyanin enhancement in plants can be achieved by knocking out negative regulators of anthocyanin production [31,32,33,34]. Nevertheless, the enrichment of plant raw materials with various valuable substances through genome editing is constrained by the fact that this method has been employed mostly for the targeted disruption of genes, resulting in the loss (knockout, KO) or a quantitative reduction (knockdown) of their functions. More recently, however, it was demonstrated that genome editing can be used to enhance the function of a valuable gene. For example, restoration of *TaMYB10* gene function caused wheat grains to acquire a red color [35].

In the present study, the hypothesis that anthocyanin accumulation in the aleurone layer of barley grain is dependent upon a functional *MYC2* allele was tested by restoring the reading frame of the recessive *myc2* allele in barley with uncolored grain. The gain-of-function modification performed in the current study in the spring barley variety “Golden Promise” (GP) led to a qualitative difference from the original variety, manifested by activation of the transcription of anthocyanin biosynthesis enzymes, the presence of blue aleurone coloration, and detectable anthocyanin accumulation in the grain.

## 2. Results

### 2.1. Genotyping of Genes Controlling Anthocyanin Accumulation in cv. Golden Promise 

**Figure 1:**
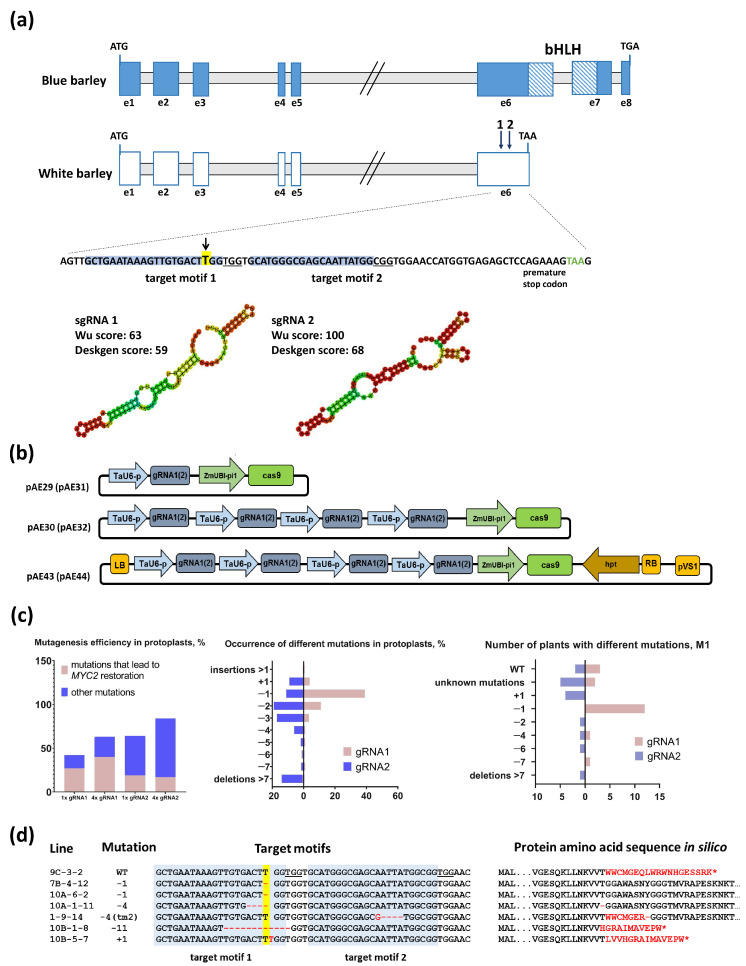
Vector construction, functional validation and mutant plants generation. (**a**) Scheme of *MYELOCYTOMATOSIS 2* (*MYC2*) gene in blue-grain barley (without “T” insertion, painted in blue) and white-grain barley (with “T” insertion, painted in white). Exons are depicted as rectangles, the bHLH domain is indicated. The premature stop codon (TAA) in white-grain barley is indicated. The target motifs for the modification of *MYC2* using RNA-guided Cas9 endonuclease (CRISPR/Cas9) are highlighted by arrows (up) and the sequences of target motifs are presented (down). A single thymidine nucleobase insertion is displayed by the arrow and highlighted in yellow. The target motifs are highlighted in blue, and the PAM sequences are underlined; gRNAs prediction scores and secondary structures are presented. (**b**) The vector architectures are based upon the CasCADE modular vector system utilized for both protoplast transfection (pAE29, pAE30, pAE31, pAE32) and *Agrobacterium*-mediated plant transformation (pAE43, pAE44). gRNA1 corresponds with target motif 1, gRNA2 with target motif 2. TaU6-p–wheat U6 promoter; gRNA–chimeric single guide RNA; ZmUBI-pi1–maize *POLYUBIQUITIN1* promoter with 5′-UTR and first intron; cas9–maize codon-optimized *cas9* endonuclease gene. (**c**) Transformation construct-dependent efficiencies of targeted mutagenesis in protoplasts after transfection with pAE29 (1× gRNA1), pAE30 (4× gRNA1), pAE31 (1× gRNA2), and pAE32 (4× gRNA2). Left diagram: the proportion of sequence reads with any mutations is represented by the whole columns and the proportion of mutations that lead to *MYC2* restoration by the pink part of the columns. The mutagenesis patterns and their frequencies obtained by both gRNAs are presented in the center. Right diagram: number of plants with different mutations in M1. (**d**) Mutation patterns of the *MYC2* mutant lines, with the nucleotide sequences shown on the left and amino acids on the right. The two target motifs for the Cas9/gRNA system are colored blue, and the PAMs are underlined. Hyphens correspond to deletions of individual nucleotides or amino acids. Differences from the fully functional protein sequence are highlighted in red. Stop codons are indicated by asterisks.

GP accumulates anthocyanins in the leaf sheath bases and nodes but has uncolored auricles and grains (Figure S1). The ability to accumulate anthocyanins in different organs indicates that the structural genes encoding the enzymes of anthocyanin biosynthesis are functional. Hence, all differences in tissue-specific distribution of anthocyanins lie in the regulatory mechanisms. Jia et al. [11] described variations of MbHF35 cluster gene sequences typical for blue and uncolored barley. We analyzed MbHF35 cluster gene sequences from GP and showed that variations in all three genes correspond to white grain barley genotypes (Table S1, Figure S2a). Additionally, we found that GP has the H4 haplotype of *GST*, while there are blue grain barley genotypes with H4 haplotype ([13], Table S1). It was concluded that the critical mutation determining white grain color in GP is the single-nucleotide insertion in *MYC2*. The full-length MYC2 protein contains 560 amino acid residues and includes a bHLH DNA-binding domain located between the amino acid residues 382 and 444 (Figure S2b). The insertion results in a reading frame shift after 361 amino acids along with the generation of a premature stop codon 19 amino acids downstream of the mutation site (Figure 1a) and upstream of the functional bHLH domain. Accordingly, it was hypothesized that restoration of MYC2 function could activate anthocyanin accumulation in the aleurone layer of the grain in GP.

### 2.2. Development of Genetic Constructs for Efficient MYC2 Reading Frame Restoration

The canonical cleavage site of one selected Cas9/gRNA target motif perfectly matches the position of the thymidine base that abolishes the gene function (Figure 1a). The cognate gRNA, referred to as gRNA1, exhibited a reasonably acceptable activity prediction score but a suboptimal predominant secondary structure (Figure 1a, Table S2). A second target motif was selected in close proximity, exhibiting superior predicted activity and a very suitable secondary structure of its corresponding gRNA, designated as gRNA2 (Figure 1a, Table S2). To ascertain whether the efficiency in inducing precise modifications is dependent upon the gRNA gene dosage, vectors containing either one or four identical gRNA expression cassettes were generated for each gRNA (Figure 1b).

For testing of the activity of both designed gRNAs, a protoplast test was performed prior to stable plant transformation. Two possible modifications were considered as potential means of restoring the reading frame: a 1-nucleotide (nt) deletion that would perfectly restore the protein structure and result in the full number of amino acids, and a 4-nt deletion leading to reading frame restoration with a single amino acid deletion. The gRNA activity test in protoplasts demonstrated that gRNA1, as expected, exhibited a lower overall efficiency than the gRNA2, yet it demonstrated that both types of desired mutations can be obtained (Figure 1c, Table S3). gRNA2 induced a high diversity of mutations, while gRNA1 predominantly caused the desired 1-nt deletions (Figure 1c). The overall efficiencies of the 1x gRNA constructs were confirmed to be lower than those of the respective 4× gRNA constructs, which applied for both gRNAs (Figure 1c, Table S3). It was shown that the construct harboring four gRNA1 copies is capable of inducing the desired modification in 40% of all mutated reads. The *cas9* and gRNA expression units from 4× gRNA vectors were transferred to the binary vectors for *Agrobacterium*-mediated transformation and utilized to generate plants edited at the *MYC2* gene.

### 2.3. Generation of Barley Lines with Restoration of the MYC2 Reading Frame

Agrobacterium-mediated transformation to GP yielded 18 and 14 primary transformants for the constructs containing gRNA1 and gRNA2, respectively. gRNA1 predominantly induced the desirable 1-bp deletion, which is consistent with the results seen in the protoplast model (Figure 1c). Such primary mutants were chosen with priority to generate segregating progeny for the selection of homozygous and T-DNA-free lines. Plants with other mutations were selected for comparison. Homozygous lines were generated through successive self-pollination of M1 to M4 plants by genotyping each generation and selection of individuals carrying various modifications in the *MYC2* (Table S4). The selection against T-DNA insertions yielded 12 non-transgenic lines. However, the transgene could not be eliminated from one line (1-9-14, Table S4).

Ultimately, seven lines representing five different genotypes were used for further phenotyping (Figure 1d, Figure S3). The lines with 1- and 4-bp deletions at target motif 1 have been designated as rMYC (restored MYC).

### 2.4. Aleurone of Lines with Restored MYC2 Takes on Blue Color

**Figure 2:**
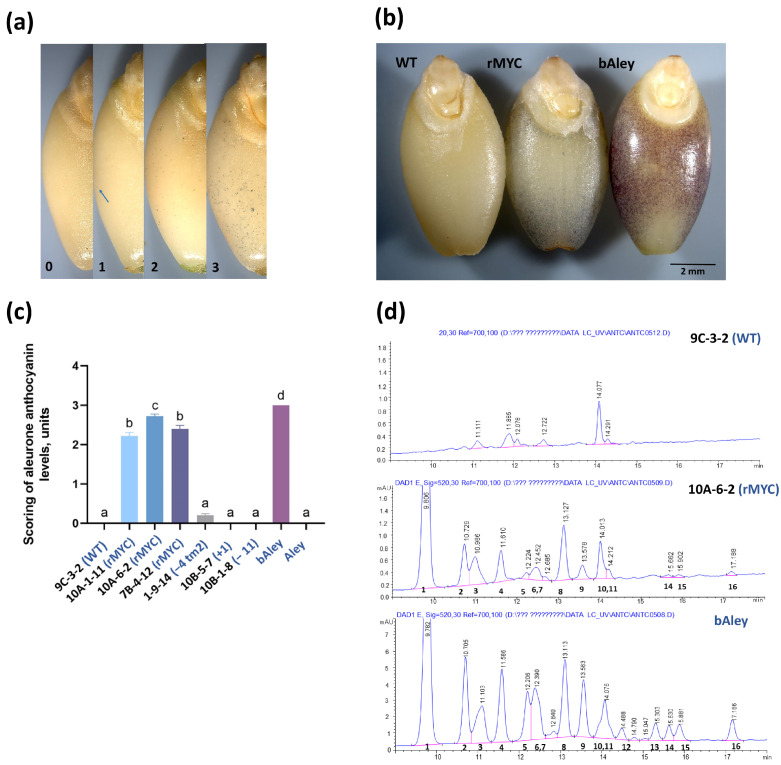
Phenotype of grains with *MYC2* reading frame restoration. Grains for all experiments were collected at the early dough stage of spike maturity (BBCH-83). (**a**) A four-level scoring system for the evaluation of anthocyanin accumulation in the aleurone layer based upon the density of blue dots; 0—complete absence of blue dots, 1—few isolated dots (dot is indicated with arrow), 2—appearance of individual dots across the entire grain surface or in small clusters, 3—sufficient number of dots to render the grain surface visibly blue (min and max are represented extremes within the range covered by this category). (**b**) Phenotype of grain from line with full *MYC2* coding sequence restoration in comparison to uncolored WT (negative control, line 9C-3-2) and grain from the natively blue barley line bAley (positive control, line 10A-6-2). (**c**) Differences in blue pigmentation as assessed using a four-level scoring in *MYC2*-edited and control homozygous lines of the M5 generation. Columns represent average scoring levels with standard error of the mean being provided as error bars. Different letters represent statistically significant differences according to ANOVA followed by Tukey’s post hoc test with Bonferroni-corrected *p*-values (*p*-value < 0.05). (**d**) Chromatograms (HPLC/UV) of anthocyanins of barley samples (λ 520 nm): wild-type—9C-3-2-3; rMYC–10A-6-2-5; positive control—bAley. The main peaks (1–16) in the chromatograms of samples 10A-6-2-5 (rMYC) and bAley represent anthocyanins (λmax range 508–530 nm). The major peak in the chromatogram of sample 9C-3-2-3(WT) with a peak release time of 14.077 min is not an anthocyanin, since it has λmax of 456 nm, which differs from λmax for anthocyanins (520 nm). The weak intensity of the remaining peaks in the chromatogram of sample 9C-3-2-3(WT) does not allow determining their group affiliation based on the maximum absorption in the UV spectrum.

Restoring the reading frame of the *MYC2* by introducing 1- or 4-bp deletions at target motif 1 led to a significant increase in the number of anthocyanin-accumulating spots in the aleurone layer of barley grains as assessed using M5 plants (Figure 2a–c). The near-isogenic line bAley with blue-colored grains and high anthocyanin content was used as the positive control [36]. The 4-bp deletion (line 10A-1-11) resulted in anthocyanin accumulation comparable to that observed in lines with perfect gene restoration (lines 7B-4-12, 10A-6-2). However, the deletion of four base pairs in the second target motif did not result in a significant increase in visible anthocyanin coloration. This may indicate the partial reconstruction of protein function by reading frame restoration combined with amino acid substitutions occurring in this genotype (Figure 1d, line 1-9-14). In contrast, in the GP control lines (WT), anthocyanin dots were completely absent (Figure 2c). Other obtained mutations, such as the deletion of 11 nucleotides or the insertion of 1 nucleotide at target site 1, did not restore the reading frame, and the aleurone remained white. Of note, the intensity of anthocyanin coloration in the rMYC grains was significantly lower than that observed in the positive control line bAley (Figure 2b).

### 2.5. Chemical Analysis Confirms Anthocyanin Accumulation in Plants with Functional MYC2

The presence of anthocyanins in the rMYC lines was confirmed by analyzing methanolic extracts of the grain of rMYC line 10A-6-2-5 and the two control lines 9C-3-2-3 (segregated WT) and bAley, using HPLC. Anthocyanins were detected in the rMYC line but not in the WT line, anthocyanin profile of rMYC line was similar to that of the blue-grained line bAley, with sixteen individual peaks differing in elution time (Figure 2d). Delphinidin-3-glucoside (Dp3Glc), cyanidin-3-glucoside (Cy3Glc), petunidin-3-glucoside, pelargonidin-3-glucoside (Pt3Glc, Pg3Glc), peonidin-3-glucoside (Pn3Glc), and malvidin-3-glucoside (Mv3Glc) were identified by MS in the anthocyanin profile of bAley (Figures S4 and S5). The anthocyanin content in the grain of the WT line was zero, while it was calculated to be 0.28 ± 0.03 mg/100g and 4.50 ± 2.17 mg/100g in the lines rMYC and bAley, respectively. This represents an almost 16-fold increase in anthocyanin content in the blue-grained line bAley compared to the rMYC line (Table S5).

Among the identified compounds, Dp3Glc was the most abundant. The second major compound identified in both anthocyanin-containing genotypes was Pn3Glc, followed by Cy3Glc, Pt3Glc, Pg3-Glc, and Mv3Glc (Table S5). The other detectable compounds were not identified, and their sum quantity comprised 15% and 21% of the total anthocyanin content in rMYC line and bAley, respectively.

### 2.6. MYC2 Restoration Does Not Affect Morphological Characteristics

Morphometric analysis was performed for modified lines during autumn and spring vegetations for M4 and M7 generations, respectively. Statistically significant differences were identified between the mutants and the wild-type lines in the M4 generation for few parameters (Table S6). No differences were found with respect to the same trait that would be reproduced in the same line across two experiments. This indicates that the observed differences are more likely due to environmental conditions rather than to the genotype.

### 2.7. Anthocyanin-Related Enzyme Genes Are Activated in Aleurone of Lines with Functional MYC2

An analysis was conducted to examine the expression of four structural genes involved in anthocyanin biosynthesis, namely *flavanone 3-hydroxylase* (*F3H*), *flavonoid 3′-hydroxylase* (*F3′H*), *flavonoid 3′,5′-hydroxylase* (*F3′5′H*), and *anthocyanidin synthase* (*ANS*), in the aleurone layer of the barley grain at the dough stage of maturity, when the synthesis and accumulation of anthocyanins is actively occurring. These genes are responsible for the final steps of anthocyanin biosynthesis and considered to be critical in this pathway [14,37,38]. The *F3H* gene was selected for expression analysis based on the results of Xu et al. [14], which showed a correlation between its expression and the transcription level of the *MYC2* gene. The *F3′5′H* and *F3′H* genes were analyzed to assess the activation of the pathway responsible for the synthesis of delphinidin and its derivatives (blue anthocyanins) and cyanidin and its derivatives (red-violet anthocyanins). The expression of the *ANS* gene was quantified because its product is responsible for the transition from uncolored anthocyanidins to colored anthocyanins, which is a crucial final reaction in the synthesis of all anthocyanin groups. In addition, the key role of this gene in the synthesis of anthocyanins in cereal grains has already been demonstrated [37].

**Figure 3:**
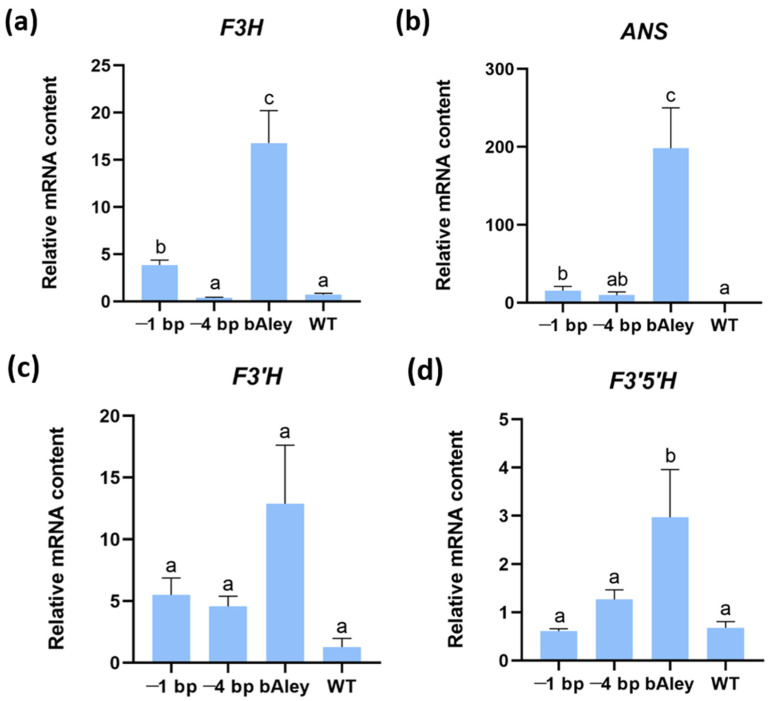
Expression of anthocyanin-related enzyme-encoding genes. (**a**–**d**): The bars represent the relative gene expression levels. The expression level of structural genes was also measured in mutants with a −4 bp deletion in the second target motif. The corresponding standard errors of the means are indicated. Exclusively different smaller case letters on top of the columns within each diagram indicate statistically significant differences (pairwise Mann–Whitney comparisons, *p*-value with Bonferroni correction for multiple testing). The designation “ab” signifies that there is no discernible distinction between the expression level and the levels designated both “a” and “b”, and that there is a statistically significant difference from the level designated “c”.

Restoration of the *MYC2* gene function resulted in 4-fold and 22-fold increases, respectively, in the expression of the *F3H* and *ANS* genes in the aleurone layer of the grain as compared to the corresponding levels in the WT (Figure 3a,b). However, these transcript levels were only approximately 23% and 8% of the expression of the same genes (*F3H*, *ANS*) in the bAley line. Conversely, no significant effect was found for the expression levels of the *F3′H* and *F3′5′H* genes (Figure 3c,d) between the rMYC line and the WT. It can therefore be postulated that the discrepancy in transcript levels of the *F3H* and *ANS* genes between the rMYC line and the bAley line may be responsible for the observed variation in grain color intensity.

## 3. Discussion

### 3.1. Using Conventional RNA-Guided Cas9 Represents an Elegant Approach to Precisely Restore Gene Function

The use of conventional Cas9 for the restoration of gene function through reading frame correction represents an elegant method that does not require complex modifications of the Cas9/gRNA system. However, this straightforward approach is applicable only for certain genetic constellations. In plant research, Zhu et al. [35] successfully restored the *TaMYB10* gene in wheat, changing grain color from white to red. A similar frameshift with gene function restoration was artificially reproduced in constructs for testing the cleavage activity of customized endonucleases [39].

In the current study, two gRNAs were tested for introducing precise modifications in the target region. The gRNA1 lacked an appropriate structure and predictably exhibited a mere moderate activity, whereas gRNA2 proved more efficient but did not perfectly address Cas9 endonuclease to the thymidine insertion that was to be removed or compensated. Consequently, it was determined that testing the constructs on protoplasts would be beneficial to assess and compare gRNA efficiencies [40,41,42,43]. It was shown that the gRNA1 not only demonstrated robust mutagenesis activity but also predominantly caused a precise single thymine base deletion in both protoplasts and plants. By contrast, gRNA2 exhibited a much broader pattern of mutations, among which there were almost no deletions useful to restore the reading frame.

The occurrence of small deletions of several base pairs at the expected Cas9 cleavage site is typically associated with the non-homologous end joining DNA repair pathway. The predominance of a specific mutation pattern is most likely related to the nucleotide environment and the presence of microhomologies [44]. It is conceivable that the deletion of one nucleotide may occur when two or more identical nucleotides flanking the expected Cas9 cleavage site are present. This is because one or two base pairs of microhomology between the two DNA termini can be utilized during a NHEJ event [45].

### 3.2. The Restoration of the MYC2 Function Leads to the Accumulation of Anthocyanins

The underlying mechanism of the blue grain trait in cereals remains poorly understood. The interaction between the *Blx* genes and their intensifiers predetermines the blue color and its intensity that can vary substantially, ranging from pink and pale blue to red and dark blue, depending on the combination of genes in dominant and recessive states, as well as the growth conditions. This variability significantly hinders the investigation of this trait [9,46], and the role of each gene component in the formation of the blue color trait needs to be identified.

In the current study, we took advantage of the regenerative capacity of the GP variety to create the precise genetic model of isogenic lines for studying the role of the *MYC2* in the anthocyanin biosynthesis activation in aleurone tissues. *MYC2* reading frame restoration in the white-grained GP resulted in the appearance of blue dots in the aleurone that were confirmed as anthocyanins by chromatography analysis (Figure 2d).

Although the quantity of anthocyanins synthesized in the aleurone layer of the rMYC line was 16 times lower than in the positive control line bAley, its anthocyanin profile was similar to this line, with the most abundant anthocyanin compound Dp3Glc accounting for 58% of the total anthocyanin content, followed by Pn3Glc (11%), Cy3Glc (9%), and Pt3Glc/Pg3-Glc (7%) that contribute from 7 to 11% to the total anthocyanin content of the grain of the rMYC line (Table S5). Both profiles are overall similar to the anthocyanin profiles with Dp3Glc and its derivatives as predominant ones identified in blue-grained *Triticeae* species [47,48], so rMYC and bAley lines should therefore be considered as blue-grained barley. Although, some curious differences were observed in the relative abundance of Mv3Glc. In the rMYC line, Mv3Glc represents only 0.7% of the total anthocyanin content, compared to an average of 9–11% of this compound in bAley and other blue-grained barley described (Table S5; [49]).

Mv3Glc, together with Pt3Glc, represents di- and mono-methylated derivatives of Dp3Glc, respectively. Unlike the blue Dp3Glc, the color of these derivatives is purple-reddish, and their abundance in the anthocyanin profiles results in the reddish color of the plant tissues. The observed relative abundance of Mv3Glc in barley is assumed to cause the purple-red color of the aleurone layer in bAley.

It can be speculated that the SNPs that were revealed in the *F3′5′H* and *HvMYB4H* genes of GP and were classified as “white” are not critical for their enzyme and TF function, respectively. However, they can affect catalytic efficiency of the enzyme or transcriptional regulation activity of the regulatory factors, which may explain the observed reduced level of the anthocyanins in the rMYC line relative to the positive control line.

### 3.3. MYC2 Acts as Part of a Complex Regulatory System

Flavonoid skeletons are formed through the activity of the flavonoid biosynthesis pathway, and diversity of the anthocyanins in plant tissues is determined by the three main reactions pathways for the synthesis of cyanidin, delphinidin, and pelargonidin and their derivatives [50]. The accumulation of the predominant delphinine-derived blue pigments in the aleurone layer of barley grain has been assumed to be regulated at the transcriptional level by the MBW complex consisting of MYC2, MYB4H, and WD40 transcription factors [12,51]. To confirm the activation of genes being known targets for the MBW complex [38], we analyzed expression of the structural genes in rMYC lines along with blue and uncolored barley. Among these genes, *F3H* and *ANS* encode enzymes that participate in anthocyanin skeleton synthesis, while the branch-specific genes *F3′5′H* and *F3′H* control delphinidin- and cyanidin-derived anthocyanin synthesis, respectively. The transcription of the *F3′5′H* and *F3′H* genes was not affected by the presence of the restored MYC2 TF at the studied developmental stage, the transcriptional levels of *F3H* and *ANS* were significantly increased in edited lines while being barely detectable in the uncolored line. [52]. It can be concluded that the MYC2 within the MBW complex is a key factor involved in the activation of anthocyanin biosynthesis in barley aleurone. Further studies are needed to investigate the mechanisms of this activation, for instance whether it is direct or mediated. The developed lines are a perfect model for further transcriptomics analyses, because they differ in the functionality of just a single TF and the observed modifications in gene expression profiles can for sure be considered as an effect of a single gene. The expression of other TFs is of particular interest, especially MYB4H, which is predicted to interact with the MYC2. The study of anthocyanin pathway gene activity at different developmental stages could precisely define the timing of the MYC2 factor action.

A question remaining to be resolved is the reason for the difference in gene expression and anthocyanin content between bAley and the rMYC lines. The observed low expression of the structural genes implies that some regulatory impairments were still present in the mutant line. This might be due to the SNPs found in the regulatory and coding regions of MBW complex genes (Table S1). Alternatively (or in addition) to this, repression of anthocyanin biosynthesis may take place in the aleurone layer of GP. A wide variety of protein or small RNA families that function in different tissues and in response to different developmental and hormonal signals have been discovered as anthocyanin accumulation repressor factors in the last decade [53]. High activity of the *F3′5′H* gene transcription in bAley is consistent with data obtained previously for the BW418 line used as donor of the blue grain trait in bAley line development [54].

As anthocyanin synthesis in plants is considered to be a part of an adaptive response to the environment, the regulatory network has evolved for fine-tuned anthocyanin biosynthesis. The MBW complex is considered to integrate plenty of stimuli, which may act in opposite directions (activate or repress anthocyanin biosynthesis) determining finally the total content of anthocyanins in certain tissues [53]. It can be speculated that unidentified GP repressors yet may interfere with the sufficient activation of anthocyanin biosynthesis structural gene expression and, consequently, anthocyanin accumulation. Further studies on the fine-tuned regulation of anthocyanin biosynthesis are required to identify intensifiers of anthocyanin biosynthesis and its repressors. This will not only help switch on the synthesis, but also intensify it as much as possible to develop new anthocyanin-rich cereals.

### 3.4. Engineering of Barley Nutritional Traits by Genome Editing

Genome editing has already made a significant contribution to the genetics of crop plants by greatly accelerating research on the genetic control of many valuable traits [55,56,57,58,59,60,61]. However, as a breeding tool, it has remained less effective than modern marker-assisted or genomic selection approaches. Nevertheless, there are traits controlled by a small number of key genes, the editing of which allows for qualitative changes in the trait without losing other properties of the variety. Correction of the *MYC2* in this work resulted in anthocyanin accumulation but did not affect grain yield in the modified plants. Further enhancements of the anthocyanin biochemical pathway and modifications in other components, such as beta-glucans, can be pursued for customized engineering of dietary properties.

## 4. Materials and Methods

### 4.1. Plant Material

The transformation experiments were conducted using the two-rowed spring-type barley (*Hordeum vulgare* L.) British cv. “Golden Promise” (GP). The donor material used for transformation and primary mutant (M1) generation were cultivated as described previously [62]. M2-M7 generations were cultivated in the hydroponic greenhouse facility of ICG SB RAS at 18–24 °C on a 16-h light/8-h dark cycle.

Near-isogenic line blue Aley (bAley) in generation BC6F6 [36] and elite barley cultivar Aley (Federal Altai Research Center of Agrobiotechnology, Barnaul, Russia) were used in grain phenotyping as controls.

For grain phenotyping and chemical and gene expression analyses, the grains of mutant lines (M5) and the control bAley line grown in pots were collected at the early dough stage of spike maturity (BBCH-83; [63]). This stage was selected because at this developmental moment, the accumulation of anthocyanins is already clearly visible, but at the same time the hull and pericarp can still be manually removed, which was required to observe the phenotype.

### 4.2. Selection of Cas9 Target Motifs and Guide RNA Design

The barley cultivar Golden Promise pseudomolecules database (BPGv2 2022) on IPK Galaxy Blast Suite (https://galaxy-web.ipk-gatersleben.de/; accessed on 1 Jule 2024)) was used to extract MbHF35 cluster gene sequences (*F3′5′H*, *MYB4H*, *MYC2*) from GP and analyze sequence variations described previously in Jia et al. [11]. In addition, sequencing of exons 6 to 8 including introns in GP was performed. The coding sequence of MYC2 (MF679157.1; [12]) was used to select the target motifs (TMs) as previously described [40]. The nucleotide sequence of the *MYC2* genomic target region in GP was confirmed by PCR and Sanger sequencing with primer pairs AE7 and AE8 (Table S7, Figure 1a, Figure S2). Guide (g) RNAs with high prediction scores and proper secondary structures were selected according to [64]. Online tools DESKGEN [65] and WU-CRISPR [66] were used to evaluate predicted activity of the selected TMs. gRNA secondary structures were modeled using the RNAfold tool (http://rna.tbi.univie.ac.at/cgi-bin/RNAWebSuite/RNAfold.cgi/; accessed on 15 October 2019) [67]). Off-target analysis was performed via BLAST of target sequences at the BARLEX BLAST (https://apex.ipk-gatersleben.de/apex/f?p=284:10; accessed on 15 October 2019) and NCBI (https://blast.ncbi.nlm.nih.gov/Blast.cgi/; accessed on 15 October 2019).

### 4.3. Generation of MYC2 Editing Constructs

The CasCADE vector system [68] was used to create constructs for *MYC2* reading frame restoration. Double-stranded DNA oligonucleotides were generated by melting and reannealing the following pairs of oligonucleotides: Myc2-1-F and Myc2-1-R (for gRNA 1), Myc2-2-F and Myc2-2-R (for gRNA 2) (Table S7). Then, each of them was cloned into vectors, containing the gRNA scaffold expression cassette under the *Triticum aestivum* U6 promoter. The pAE29, pAE30, pAE31, and pAE32 vectors containing a Zea mays codon-optimized *Spcas9* under control of the maize *POLYUBIQUITIN1* (*ZmUbi1*-*p*) promoter including 5′-UTR and first intron and gRNA expression units (one or four) were obtained during the next cloning step. At last, the cas9 and 4 gRNA expression units were transferred from the vectors pAE30 and pAE32 to the generic binary vector p6i-2 × 35s-TE9 (DNA Cloning Service, Hamburg, Germany), which resulted in the generation of the vectors pAE43 and pAE44, respectively, used for *Agrobacterium*-mediated transformation. All obtained vectors were confirmed by restriction analysis and Sanger sequencing.

### 4.4. Vector Activity Assessment in Protoplasts

The pAE29, pAE30, pAE31, and pAE32 vectors were tested by transient expression in protoplasts isolated from GP etiolated barley seedlings based on the protocol described by Gerasimova et al. [40]. Each vector was co-transformed with a pSH221-based construct containing an enhanced *GREEN FLUORESCENT PROTEIN* (e*GFP*) reporter gene [42]. Transformed protoplasts were incubated in the dark at ~23 °C for 2 days. After incubation, the transformation efficiency was estimated by calculating the proportion of GFP-positive cells using an inverted fluorescence microscope (Zeiss Axiovert 200M, filter set 13 with excitation wavelength BP 470/20 and emission wavelength BP 505–530, Figure S6). Genomic DNA was isolated from transformed protoplasts, and the target regions were amplified using primers AE1 and AE2 (Table S7). Deep sequencing of amplicons was performed by a commercial service on an Illumina MiSeq platform (https://www.novogene.com/eu-en/services/research-services/genome-sequencing/;accessed on 15 January 2020). Mutation efficiencies and patterns were calculated individually for each replicate as proportion of sequencing reads with different mutations in relation to the total number of reads including those with the wild-type sequence. The mutation frequency was normalized to the proportion of GFP-positive protoplasts representing the transformation efficiency.

### 4.5. Generation of Homozygous Lines with MYC2 Reading Frame Restoration

The binary vectors pAE43 and pAE44 were transferred by electroporation into *A. tumefaciens* strain AGL1. *Agrobacterium*-mediated gene transfer to immature embryos (GP wild-type) was performed following a method described previously [69]. The plants were analyzed for the presence of *cas9* and the selection marker *hygromycin phosphotransferase* (*hpt*) transgene by PCR using primers Cas9F and Cas9R or HygF and HygR, respectively (Table S7). The M1 plants were screened for mutations in the *MYC2* gene by PCR amplification of the target regions using primers AE7 and AE8 (Table S7) followed by Sanger sequencing of the amplicons. Sanger chromatograms were analyzed for the presence of nucleotide sequence changes or abnormalities in the target motif. Progeny (M2-M4) of primary mutant plants were grown under standard greenhouse conditions (16 h, 18 °C/8 h, 16 °C). Samples for sequencing were prepared by extracting genomic DNA and conducting PCR using primers Myc2_3_F and Myc2_30_R (Table S7). Deep sequencing was performed on the Illumina MiSeq platform in the Shared Facility Center for Genomic Research at the ICG SBRAS (Novosibirsk, Russia). The obtained nucleotide sequences were aligned to the reference sequence in order to estimate the ratio of reads with different mutations. Sanger sequencing was utilized for genotyping of M5 plants, and for few selected individuals from other generations as confirmation and verification of genotypes.

### 4.6. Phenotyping of Grains

During the assessment of anthocyanin accumulation in the aleurone layer, an original method specifically developed for this experiment was utilized. It was observed that the blue pigmentation in both the bAley barley line and in rMYC2 lines develops during the dough stage of grain maturity, manifesting as varying amounts of colored spots on the surface of the aleurone layer. These spots exhibit blue or violet hues, forming different patterns of distribution. Presumably, each colored spot corresponds to an individual cell where anthocyanin synthesis was independently activated. To evaluate the degree of manifestation of the pigmented aleurone phenotype, plants were grown under identical conditions until the dough stage of grain maturity. Three spikes were selected from each plant, and ten grains were taken from each spike. The layers of the grains above the aleurone layer were then manually removed. Using a stereomicroscope, the density of colored spots on each grain was assessed individually. A four-level numerical scale was employed, assigning values from 0 to 3 to each grain: 0—no spots, 1—single spots, 2—a cluster of blue spots, 3—a large number of blue spots (Figure 2a). These data were compiled into a unified table that was subsequently used for analysis and examination of differences between genotypes. Statistical analysis was performed using ANOVA and Tukey’s post hoc test with Bonferroni corrected *p*-values. Differences between groups were considered significant if the *p*-value was less than 0.05.

### 4.7. Gene Expression Analysis

To prepare samples for RNA isolation, barley grains at the requisite stage were peeled from lemma, palea, and pericarp tissues. The embryos were excised and removed, while the aleurone layers were carefully scraped off the grains to avoid capturing the starchy endosperm and immediately frozen in liquid nitrogen. The RNA was extracted using the RNeasy Plant Mini Kit (Qiagen, Hilden, Germany) and the resultant samples were treated with DNase (Thermo TF, Foster City, CA, USA). cDNA was obtained using the RevertAid First Strand cDNA Synthesis Kit (Thermo TF, USA). Real-time PCR was performed using the BioMaster HS-qPCR Lo-ROX SYBR kit (Biolabmix, Novosibirsk, Russia) on a QuantStudio5 instrument (Thermo TF, USA). For each genotype, reactions were carried out on samples from five biological replicates, with each three technical replicates. The transcription levels were calculated relative to the expression of two reference genes, *ACTIN* (*ACT*) and *MALATE DEHYDROGENASE* (*MDH*), which had previously been shown to be stable in barley grain tissue [70].

The relative expression levels were calculated using the delta Ct method [71]. The gene expression between genotypes was compared using the nonparametric Kruskal–Wallis analysis of variance of Ct values of different groups for the corresponding gene and pairwise Mann–Whitney comparisons, in which the *p*-value was taken with Bonferroni correction for multiple testing (Past, version 4.03). Significant differences were identified at *p*-values of less than 0.05.

### 4.8. Extraction and Chromatographic Analysis of Anthocyanins

To concentrate anthocyanins accumulated in the aleurone layer, the grains were peeled out of the lemma, palea, pericarp, and seed coat tissues, the embryo was removed, and the peeled embryo-less grains were dried in vacuum. For each genotype, grains from two individual plants were collected separately and analyzed independently. From each plant, 30 grains were collected from three spikes with 10 grains per spike. Then, the dried grains were ground up and used for anthocyanin extraction. A ground barley grain sample (~400 mg) was extracted with 2 × 4 mL of MeOH-1N HCl (85:15) using an ultrasonic bath at 55 °C. The extracts were combined, centrifuged, and the supernatant liquid was evaporated at 50 °C to a volume of ~0.5 mL. To remove the interfering influence, the resulting extract was passed through a layer of Diasorb C18 sorbent. The fraction of colored compounds was collected and analyzed by high-performance liquid chromatography with diode array and mass-selective detectors (Methods S1 and S2).

### 4.9. Morphometric Analysis

An analysis of morphometric characteristics was carried out on 6 barley lines of the M4 and M7 generations from a greenhouse. In total, 11 characteristics were investigated: general bushiness (total number of stems), productive bushiness (number of stems bearing spikes), plant height, awn length, main (largest) spike length, average spike length, density of spikelets on the main spike, number of grains in the main spike, weight of grains from the main spike, number of grains per spike, weight of grains per plant. In order to calculate the average spike length, all well-developed spikes were taken into account, provided that there were up to 10 of them on the plant. In the event that the number of mature, well-developed spikes exceeded 10, 10 of them were randomly selected and measured.

Measurements were made on 10 plants of each genotype at full maturity (at harvest). The means and standard deviations were calculated in Microsoft Excel for all the parameters of each line. The significance test was done using the program STATISTICA v. 6. A non-parametric analysis of variance was performed using the Kruskal–Wallis test, followed by Dunn’s post hoc test with Bonferroni correction for multiple testing. Based on the results of pairwise comparisons, differences between groups were considered significant with a *p*-value < 0.05.

## 5. Conclusions

Successful reading frame restoration of the *MYC2* gene in barley cv. Golden Promise allowed us to generate isogenic lines serving as unique genetic model to study the regulation of anthocyanin biosynthesis and to show the power of precise directed genome modifications for gene activation and trait design. The obtained mutant lines with restored MYC2 reading frame demonstrated a visible colored aleurone phenotype, accumulation of a spectrum of anthocyanins in grains and the activation of structural genes of the anthocyanin biosynthesis pathway. The critical role of the MYC2 transcription factor for the blue aleurone trait was unequivocally demonstrated, confirming previous findings and providing a biotechnological solution for the enrichment of barley grain with valuable dietary components. The isogenic lines developed in the study can further be used for different sorts of analyses aiming to precisely investigate the molecular function of MYC2.

## Data Availability

The original contributions presented in the study are included in the Supplementary Material; further inquiries can be directed to the corresponding author.

## Supplementary Materials

The following supporting information can be downloaded at: https://www.mdpi.com/article/10.3390/ijms252312705/s1. References [11,13,54,70,72,73] are cited in the Supplementary Materials.
