# Supplementary material for: Accumulation of Anthocyanin in the Aleurone of Barley Grains by Targeted Restoration of the MYC2 Gene
Source: Int J Mol Sci. 2024 Nov 26;25(23):12705. doi: 10.3390/ijms252312705 (PMC11641404; doi:10.3390/ijms252312705)
Supplement: Supplementary file 1 [file ijms-25-12705-s001.zip › ijms-3285324-supplementary.pdf]

International Journal of
Molecular Sciences

MDPI

Supporting information

Table S1. Sequence variations for *F3'5'H*, *MYB4H, MYC2* and *GST* in GP.
Table S2. The structure and predicted activity of guide RNAs
Table S3. Assessment the gRNA efficiency in protoplasts
Table S4. Selection of genotypes during homozygous line generation
Table S5. Quantity of the individual anthocyanins in barley grain extracts of the line 10A-6-2 (-1) and bAley.
Table S6. Mean values of morphometric characteristics with the corresponding standard deviation obtained in generations M4 and M7 plants.
Table S7. Primers and oligos used in the study.
Figure S1. Leaf sheath bases, node, auricle, spike, and grain of GP plants grown in the greenhouse.
Figure S2. MYC2 sequences.

Figure S3. Sanger sequencing of the target motifs of the *MYC2* gene in M5 plants.

Figure S4 Mass spectrum of a total barley extract sample bAley.

Figure S5. Mass spectra of the Cy3Glc (a) and Dp3Glc (b) in barley grain extracts.

Figure S6. Identification of transformed protoplasts via GFP expression.

Method S1. High-performance liquid chromatography with diode array
Method S2. High-performance liquid chromatography with mass-selective detector.

Supplemental tables

Table S1. Sequence variations for *F3'5'H*, *MYB4H, MYC2* and *GST* in GP.

Gene	Sequence variation in GP (Position in the gene and nucleotide sequence															Haplotype	Grain color of varieties with this haplotype	Reference
MYC2	+256	+354	+506	+587	+1123	+1283	+1351	+1362	+1577	+1910	+1970	+2544	+2504	+2914	+3126	«White»	White	[11]
	T	A	A	G	A	C	C	G	TA-TATA	C	G	C	T	T	C			
MYB4H	+342	+483	+780	3'UTR												«White»	White	
				+9		+210	+216	+305	+315	+349	+351		+391	+414	+437			
	C	G	G	2bp del		C	A	G	A	T	4bp del		C	T	A			
F3'5'H	-925			-829		-808	-609	-63	+4	+686	+717	+768	+831	+917	+1141	«White»	White	
	AGGTATC			1bp del		T	A	T	G	T	C	A	C	C	C			
GST	-1224	-1124	-998	-980	-905	-786	-705	-691	-652	-465	-457	-447	-355	-257	-238	H4	White and blue	[13]
	T	T	T	A	A	T	A	del	A	A	A	T	A	C	del			

Table S2. The structure and predicted activity of guide RNAs.

	Target motif with PAM	gRNA	Strain	Wu crispr score	DeskGen score	GC %	BLAST
**Myc2-1**	gaataaagttgtgacttggtgg	gaataaagttgtgacttgg	+	63	59	35	Ok
**Myc2-2**	gcatgggcgag-caattatggcgg	gcatgggcgagcaattatgg	+	100	68	55	Ok

Table S3. Assessment the gRNA efficiency in protoplasts

Construct	Number of analyzed reads	Transformation efficiency	Overall mutation frequency	Goal mutation (-1 or -4) frequency
**pAE29 (gRNA 1; 1x)**	807	93 %	42%	27%
**pAE30 (gRNA 1; 4x)**	450	92 %	63%	40%
**pAE31 (gRNA 2; 1x)**	346	90 %	64%	19%
**pAE32 (gRNA 2; 4x)**	354	91 %	84%	17%

Table S4. Selection of genotypes during homozygous line generation

M1		M2		M3		M4		site
**plant**	**genotype**	**plant**	**genotype**	**plant**	**genotype**	**genotype**	**transgene**	
**10A**	-1/-4	10A-1	-4	10A-1-11	-4	-4	-	1
		10A-2	-1	10A-2-6	-1	-1	-	
		10A-6	-1	10A-6-2	-1	-1	-	
		10A-9	-4	10A-9-9	-4	-4	-	
**10B**	chimera/-1	10B-1	-11/-3/-2/-1	10B-1-8	-11/-4/-1	-11	-	

		10B-5	+1	10B-5-7	+1/WT	+1	-	
**7B**	WT/-1	7B-1	WT	7B-1-1	WT	WT	-	
		7B-2	-1	7B-2-2	-1	-1	-	
		7B-4	-1	7B-4-12	-1	-1	-	
**9C**	WT	9C-3	WT	9C-3-2	WT	WT	-	
**1**	chimera/-3	1-9	-4/-3/-2	1-9-1	-4/-5	-4	-	2
				1-9-2	-4	-4	-	
				1-9-14	-4	-4	+	

Table S5. Quantity of the individual anthocyanins in barley grain extracts of the line 10A-6-2 (-1) and bAley, mg Dp3Glc/100g.

**Characteristics of chromatogram peaks**				**Quantity of the anthocyanins in barley grain extracts, mg/100g**							
**Peak**	**Compound**	**T, min**	**λmax, nm**	**10A-6-2**				**bAley**			
				**Replicate**		**Mean±SD**	**Part of total, %**	**Replicate**		**Mean±SD**	**Part of total, %**
				**1**	**2**			**1**	**2**		
**1**	Dp3Glc	9.92	526	0.166	0.163	0.164 ±0.002	58.037	0.976	2.443	1.710±1.037	37.966
**2**	Cy3Glc	10.83	518	0.022	0.028	0.025±0.004	8.952	0.266	0.708	0.487±0.313	10.821
**3**	Pt3Glc Pg3Glc	11.22	526	0.023	0.015	0.019±0.006	6.783	0.205	0.716	0.461±0.361	10.227
**4**	Pn3Glc	11.70	526	0.017	0.047	0.032±0.021	11.194	0.246	0.746	0.496±0.354	11.006
**5**	Mv3Glc	12.33	524	0.003	0.001	0.002±0.001	0.735	0.162	0.663	0.413±0.355	9.161
**6**	u.a.	12.53	524	0.010	0.003	0.007±0.005	2.311	0.122	0.152	0.137±0.021	3.047
**7**	u.a.	12.53	524	0.002	0.003	0.002±0.001	0.877	0.122	0.152	0.137±0.021	3.047
**8**	u.a.	13.23	530	0.029	0.000	0.015±0.021	5.127	0.251	0.050	0.150±0.143	3.339
**9**	u.a.	13.68	508	0.008	0.000	0.004±0.006	1.435	0.179	0.064	0.121±0.081	2.693
**10**	u.a.	14.19	518	0.018	0.000	0.009±0.013	3.153	0.087	0.199	0.143±0.079	3.174
**11**	u.a.	14.19	522	0.004	0.000	0.002±0.003	0.700	0.087	0.099	0.093±0.009	2.068
**12**	u.a.	14.60	528	0.000	0.000	0.000±0.000	0.000	0.037	0.000	0.019±0.026	0.416
**13**	u.a.	15.42	522	0.000	0.000	0.000±0.000	0.000	0.057	0.022	0.040±0.025	0.879
**14**	u.a.	15.74	522	0.002	0.000	0.001±0.001	0.353	0.045	0.025	0.035±0.014	0.772
**15**	u.a.	15.99	522	0.002	0.000	0.001±0.001	0.353	0.058	0.000	0.029±0.041	0.641
**16**	u.a.	17.28	522	0.000	0.002	0.001±0.001	0.314	0.067	0.000	0.033±0.047	0.743
**Total (peaks 1-16)**	-	-	-	0.304	0.262	0.283±0.030	100.000	2.97	6.04	4.503±2.172	100.000

u.a. – unidentifed anthocyanins

Table S6. Mean values of morphometric characteristics with the corresponding standard deviation obtained in generations M4 and M7 plants.

**Trait\line**	**9C-3-2 (WT)**		**10A-1-11 (-4, TM1)**		**10A-6-2 (-1, TM1)**		**7B-4-12 (-1, TM1)**		**1-9-14 (-4, TM2)**		**10B-1-8 (-11, TM1)**	
	**M4**	**M7**	**M4**	**M7**	**M4**	**M7**	**M4**	**M7**	**M4**	**M7**	**M4**	**M7**
**Total bushiness**	9,2 ± 2,4	15,2 ± 2,5	11,4 ± 2,1	11,3 ± 2,8	12,6 ± 1,7	10,6 ± 1,7	18,9 ± 4,4*	13,3 ± 4,6	11,4 ± 3,0	8,7 ± 2*	9,4 ± 1,8	11,9 ± 2,1
**Productive bushiness**	8,5 ± 2,0	7,5 ± 2	9,2 ± 1,4	6,6 ± 1,6	11,1 ± 1,4	6,9 ± 1,5	17,3 ± 3,5*	9,2 ± 2,3	10,7 ± 2,5	6,9 ± 1,8	7,2 ± 2,3	9,4 ± 1,8
**Plant height, cm**	62,9 ±	55,6 ±	64,6 ±	60 ±	65,1 ±	56,9 ±	63,8 ±	57,4 ±	63,9 ±	58,2 ±	69,9 ±	55,6 ±

	2,7	2	2,8	2,6*	2,6	2,7	2,1	1,6	1,3	3,7	3,1*	1,9
**Length of awns, cm**	7,9 ± 0,6	8 ± 1	7,6 ± 0,7	8,3 ± 0,4	7,6 ± 0,9	8,4 ± 0,4	8,4 ± 0,7	8,5 ± 0,4	8,1 ± 0,7	8,6 ± 0,5	7,6 ± 0,9	8,1 ± 0,8
**Main spike length, cm**	8,9 ± 0,5	8,2 ± 0,9	8,7 ± 0,7	9,7 ± 0,5*	7,7 ± 1,1	9,3 ± 0,4	9,1 ± 0,6	9,1 ± 0,8	8,3 ± 0,5	8,8 ± 1,2	9,3 ± 0,8	8,8 ± 0,7
**Average spikelet length**	6,7 ± 0,4	7,2 ± 0,5	6,9 ± 0,4	7,3 ± 0,6	6,7 ± 0,5	6,9 ± 0,5	7,3 ± 0,4	6,9 ± 0,6	6,6 ± 0,2	7,4 ± 0,5	7,5 ± 0,6	6,7 ± 0,3
**Spikelet density on the main spike**	7,8 ± 1,1	10,3 ± 1,3	7,4 ± 1,7	10 ± 1,8	10,3 ± 2*	8,2 ± 2	8,2 ± 1,2	9 ± 1,9	7,1 ± 1,2	8,4 ± 2,4	7,2 ± 2,4	10 ± 2,5
**Number of grains in the main spike, pcs**	16,2 ± 2,2	15,5 ± 3,1	13,2 ± 3,2	18,4 ± 3,4	15,3 ± 3	12,9 ± 3,9	15,7 ± 3,6	14,1 ± 3,6	11,7 ± 2,9	12,6 ± 3,6	13,9 ± 7,3	15,4 ± 5,5
**Weight of grains from the main spike, g**	0,6 ± 0,1	0,67 ± 0,12	0,5 ± 0,1	0,86 ± 0,18	0,5 ± 0,2	0,6 ± 0,16	0,6 ± 0,2	0,67 ± 0,16	0,5 ± 0,1	0,59 ± 0,15	0,5 ± 0,2	0,66 ± 0,2
**Number of grains per plant**	110,8 ± 23,4	82,6 ± 25,4	101,4 ± 19,7	89,7 ± 20,8	131,4 ± 38,2	70,9 ± 15,5	229,6 ± 43,5*	90,2 ± 17,2	137,2 ± 36,2	79,4 ± 28,9	87,3 ± 53,6	104 ± 32,4
**Weight of grains per plant, g**	3,9 ± 0,8	3,41 ± 1,01	3,9 ± 0,9	4,12 ± 0,89	4,1 ± 1,2	3,03 ± 0,72	8,7 ± 2,5*	4,24 ± 0,82	4,9 ± 1,6	2,91 ± 0,75	2,9 ± 1,6	4,27 ± 1,29

M4 *Differences between mutant and WT lines were significant (Dunn's test, p = 0,00000381 - 0,04924)
M7 *Differences between mutant and WT line were significant (Dunn's test, p = 0,000481 - 0,004964)

Table S7. Primers and oligos used in the study.

Name	Sequence 5‘-3‘	Application	Reference
**Myc2_1_F**	CTTGGAATAAAGTTGTGACTTGG	Cloning, introduction of gRNAs in vectors	
**Myc2_1_R**	AAACCCAAGTCACAACTTTATTC		
**Myc2_2_F**	CTTGGCATGGGCGAGCAATTATGG		
**Myc2_2_R**	AAACCCATAATTGCTCGCCCATGC		
**AE7**	TCTGGGAGCTGCACTTGC	Primary mutant plants screening	
**AE8**	CGGGCACTTTACCTCCAACA		
**AE1**	GAAGGCTCTCGTCCATCC	Protoplasts and mutant plants screening	
**AE2**	CGTCTTCTCTCTGCCATGA		
**Myc_3_F**	TTAGGCGAAGGCTCTCGTCCATCC	Mutant plants screening	
**Myc_30_R**	CACCGGCGTCTTCTCTCTGCCATGA		
**HygF**	CATGGTGGAGCACGACACTCTC	Transgene screening	
**HygR**	GATTCCTTGCGGTCCGAATG		
**Cas9F**	TTTAGCCCTGCCTTCATACG		
**Cas9R**	TTAATCATGTGGGCCAGAGC		
**F3H_RT_F**	CACCATAACGCTCCTCCT	Real-time PCR for *F3H* gene	[72]
**F3H_RT_R**	GCCTGCTGCTCTCCC		
**F3’H-1_RT_F**	GCCAGGGAGTTCAAGGACA	Real-time PCR for *F3’H* gene	[54]
**F3’H-1_RT_R**	CTCGCTGATGAATCCGTCCA		
**F3’5’H-1_RT_F**	ATCGCATGTCGTGGCTATG	Real-time PCR for *F3‘5’H* gene	[54]
**F3’5’H-1_RT_R**	GCCGAGTTCACCATCATTTC		
**ANS_RT_F**	CTTGCCGATCGAGGACAAG	Real-time PCR for *ANS* gene	[72]
**ANS_RT_R**	GTGGAGCATGTGGAACAAATAG		
**HvMDH_RT_F**	GCACTGGTGTGAATGTTGC	Real-time PCR for *MDH* gene	[70]
**HvMDH_RT_R**	CTTCTCAGGGATAGATGGAGC		
**HvActin.001**	TCGCAACTTAGAAGCACTTCCG		[73]

**HvActin.002**	AAGTACAGTGTCTGGATTGGAGGG	Real-time PCR for *Actin* gene	

Supplemental figures

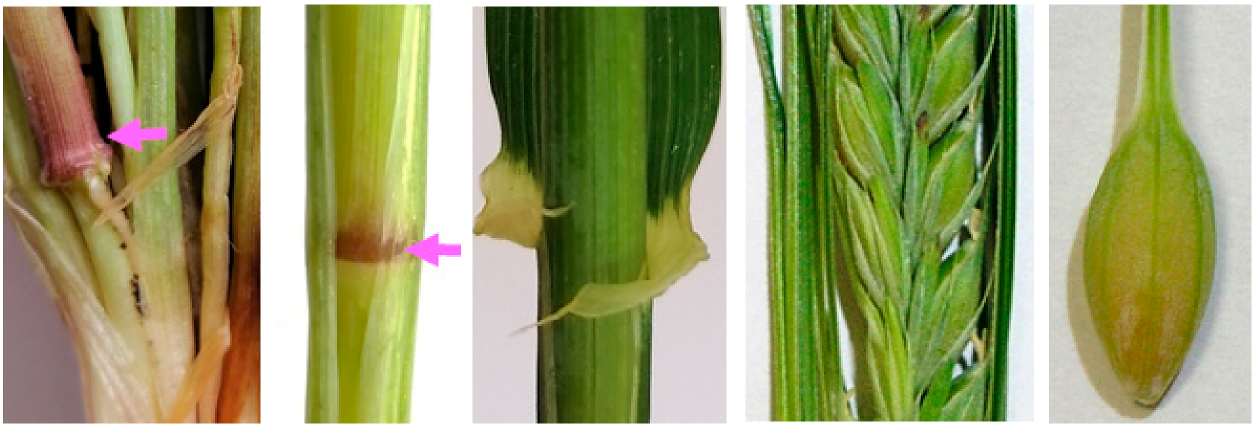

Figure S1. Leaf sheath bases, node, auricle, spike, and grain (from left to right) of GP plants grown in the greenhouse (autumn vegetation season). Anthocyanins accumulating in parts of barley plants are shown by magenta arrows.

(a)

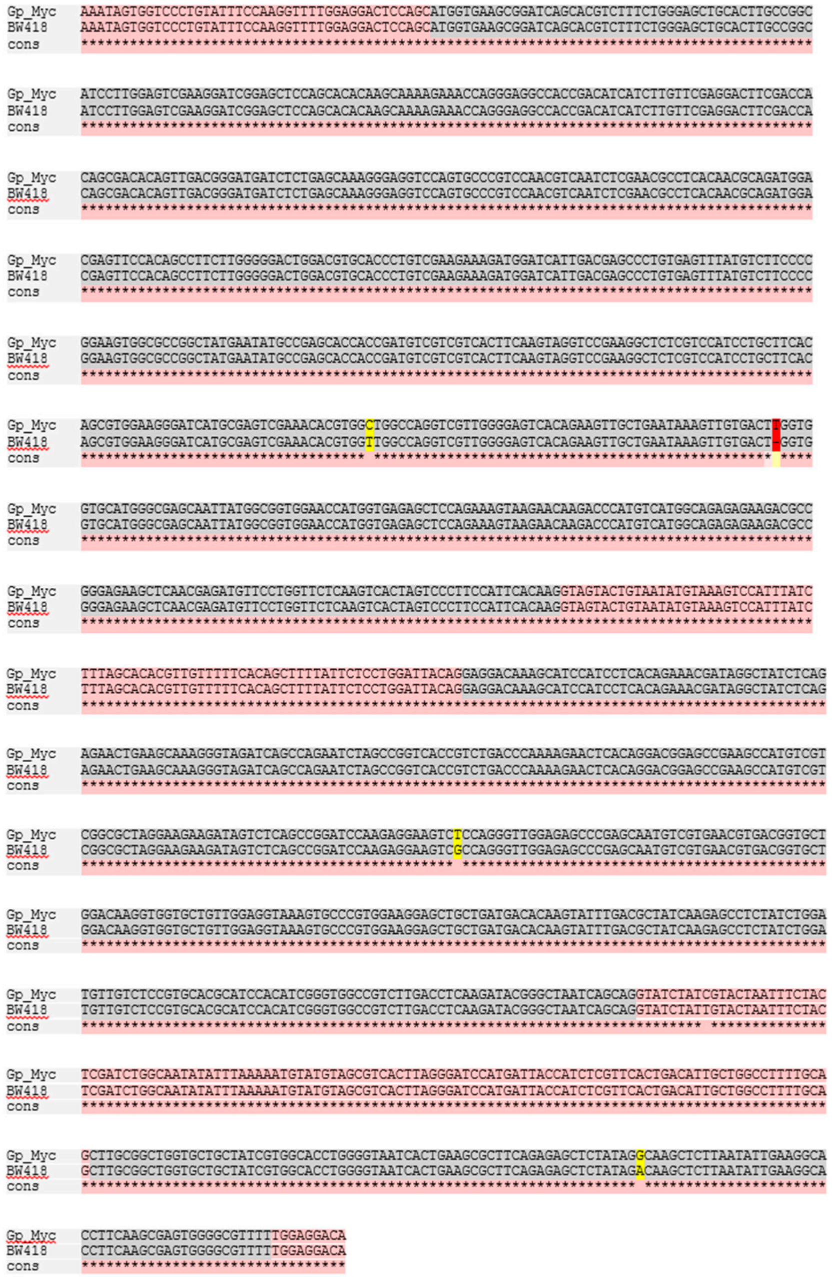

(b)

GP

MALSAPPSQEQPSGKKFGYHLAAAVRSINWTYGIFWSISASPRPGHSSVLTWKDGFYNGEIKTRKITGSTTPEFTPDERV
MHRSKQLRQLYESLLPGNSDQRARRCAASLSPEDLGDGEWYYTISMTYKFHLNQGLPGKSFASNQYVWLCNAQNAST
RTFPRALLAKVTFFQTIVCIPFMGGVLELGTLDQVLEDSSMVKRISTSFWELHLPASLESKDRSSSTQAKETREATDIILFED
FDHSDTVDGMISEQREVQCPSNVNLERLTTQMDEFHSLLGGLDVHPVEERWIIDEPCEFMSSPEVAPAMNMPSTTDV
VVTSSRSEGSRPSCFTAWKGSCESKHVAGQVVGESQKLLNKVVTWWCMGEQLWRWNHGESSRK-

GP (restored reading frame)

MALSAPPSQEQPSGKKFGYHLAAAVRSINWTYGIFWSISASPRPGHSSVLTWKDGFYNGEIKTRKITGSTTPEFTPDERV
MHRSKQLRQLYESLLPGNSDQRARRCAASLSPEDLGDGEWYYTISMTYKFHLNQGLPGKSFASNQYVWLCNAQNAST
RTFPRALLAKVTFFQTIVCIPFMGGVLELGTLDQVLEDSSMVKRISTSFWELHLPASLESKDRSSSTQAKETREATDIILFED
FDHSDTVDGMISEQREVQCPSNVNLERLTTQMDEFHSLLGGLDVHPVEERWIIDEPCEFMSSPEVAPAMNMPSTTDV
VVTSSRSEGSRPSCFTAWKGSCESKHVAGQVVGESQKLLNKVVTGGAWASNYGGGTMVRAPESKNKTHVMAERRR
REKLNEMFLVLKSLVPSIHKEDKASILTETIGYLRELKQRVDQPESSRSPSDPKELTGRSRSHVVGARKKIVSAGSKRKSPGL
ESPSNVVNVTVLDKVVLLEVKCPWKELLMTQVFDAIKSLYLDVVSVHASTSGGRLDLKIRANQQLAAGAAIVAPGVITEA
LQRAL-

BW418

MALSAPPSQEQPSGKKFGYHLAAAVRSINWTYGIFWSISASPRPGHSSVLTWKDGFYNGEIKTRKITGSTTPEFTPDERV
MHRSKQLRQLYESLLPGNSDQRARRCAASLSPEDLGDGEWYYTISMTYKFHLNQGLPGKSFASNQYVWLCNAQNAST
RTFPRALLAKVTFFQTIVCIPFMGGVLELGTLDQVLEDSSMVKRISTSFWELHLPASLESKDRSSSTQAKETREATDIILFED
FDHSDTVDGMISE[illegible]SNVNLERLTTQMDEFHSLLGGLDVHPVEERWIIDEPCEFMSSPEVAPAMNMPSTTDV
VVTSSRSEGSRPSCFTAWKGSCESKHVVGQVVGESQKLLNKVVTGGAWASNYGGGTMVRAPESKNKTHVMAERRR
REKLNEMFLVLKSLVPSIHKEDKASILTETIGYLRELKQRVDQPESSRSPSDPKELTGRSRSHVVGARKKIVSAGSKRKSPGL
ESPSNVVNVTVLDKVVLLEVKCPWKELLMTQVFDAIKSLYLDVVSVHASTSGGRLDLKIRANQQLAAGAAIVAPGVITEA
LQRAL-

Figure S2. MYC2 sequences. (a) *MYC2* gene sequences in GP and BW418 (exons 6-8). Exons are highlighted in gray, introns in red, SNPs in yellow, "T" insertion in red. The first SNP changes the amino acid valine (BA) to alanine (GP); the other two SNPs do not affect the protein sequence. (b) Protein sequences in GP and BA. V to A substitution is highlighted in yellow, bHLH domain is highlighted in red. The incorrect amino acids resulting from the single nucleotide insertion are underlined.

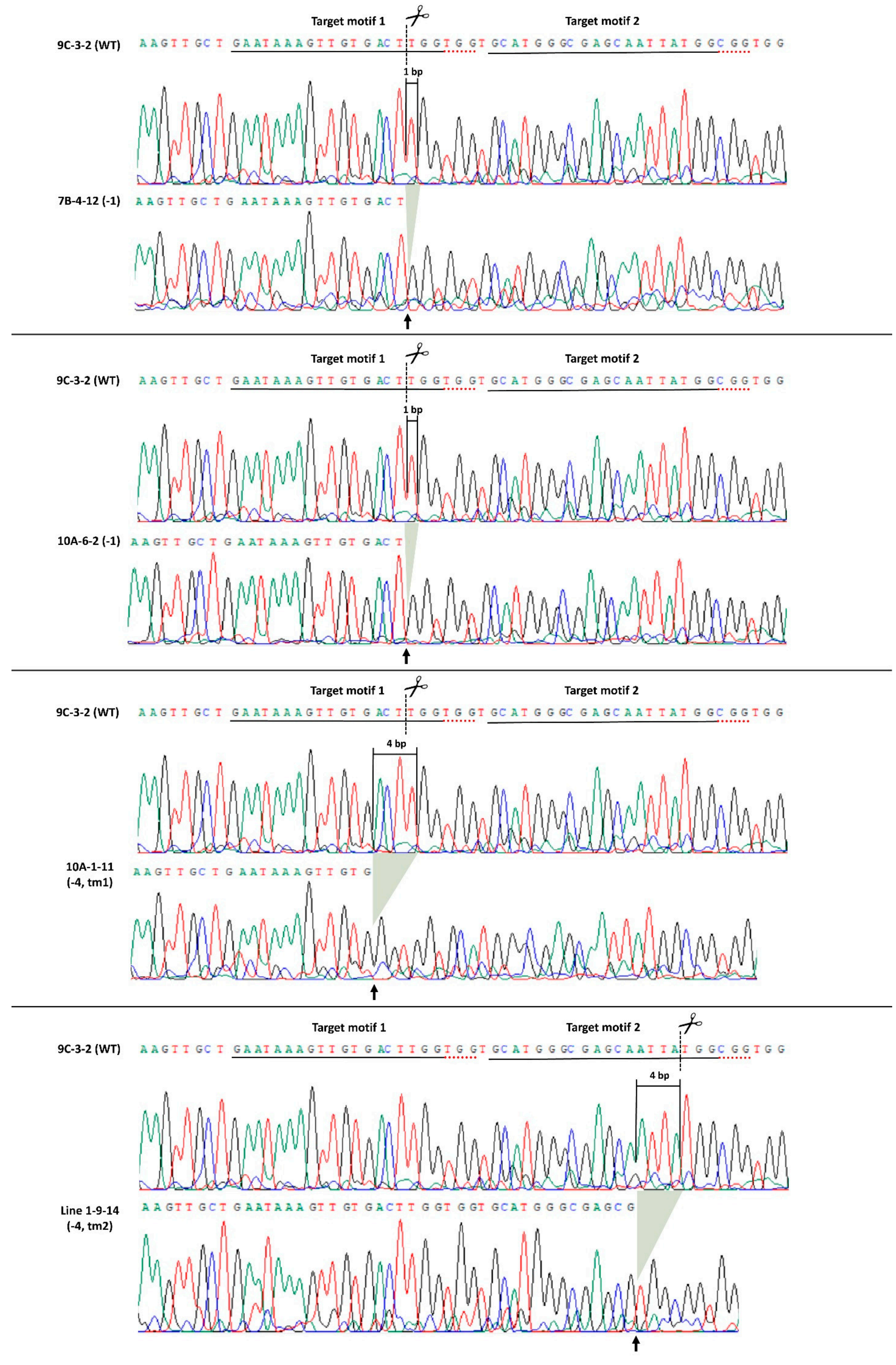
Target motif 1
Target motif 2
9C-3-2 (WT)
AAGTTGCT GAATAAAGTTGTGACTTGGTGGTGCATGGGCGAGCAATTATGGCGGTGG
1 bp
7B-4-12 (-1)
AAGTTGCTGAATAAAGTTGTGACT
Target motif 1
Target motif 2
9C-3-2 (WT)
1 bp
10A-6-2 (-1)
AAGTTGCTGAATAAAGTTGTGACT
Target motif 1
Target motif 2
9C-3-2 (WT)
4 bp
10A-1-11
(-4, tm1)
AAGTTGCTGAATAAAGTTGTG
Target motif 1
Target motif 2
9C-3-2 (WT)
4 bp
Line 1-9-14
(-4, tm2)
AAGTTGCTGAATAAAGTTGTGACTTGGTGGTGCATGGGCGAGCG

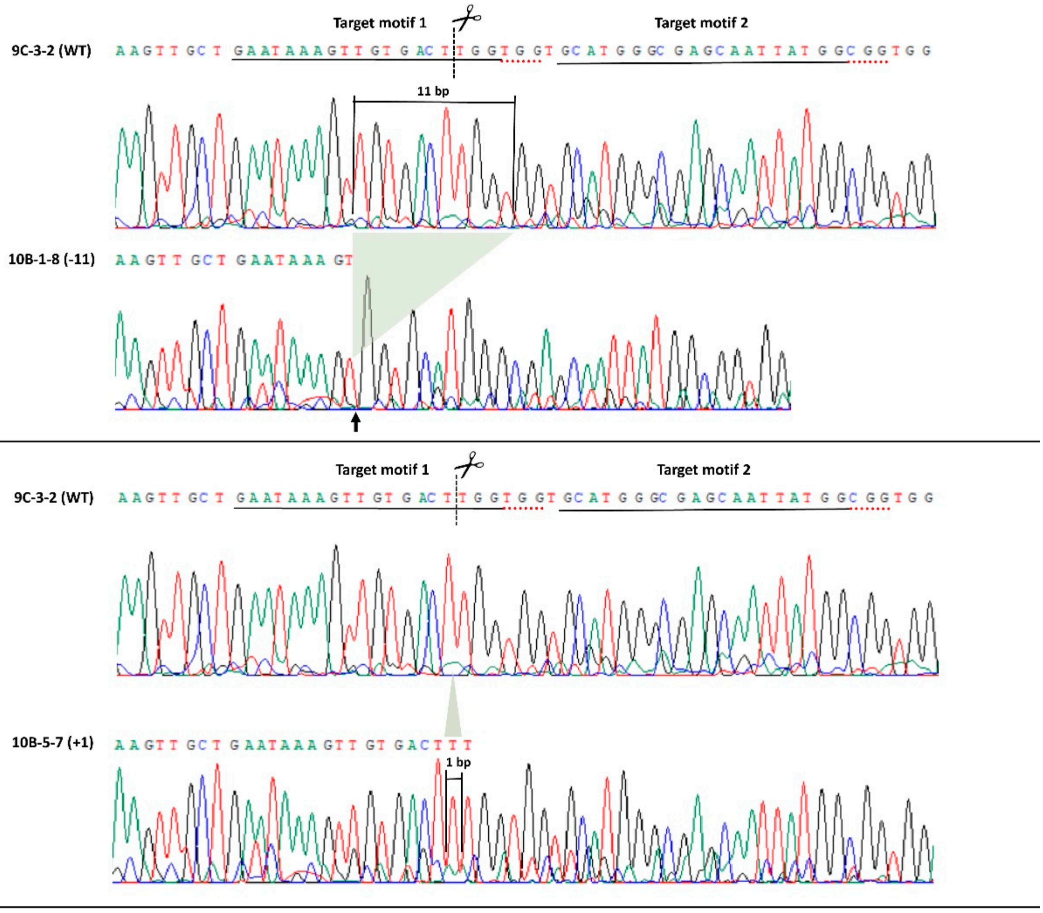

Figure S3. Sanger sequencing of the target motifs of the MYC2 gene in M5 plants. The Sanger chromatograms of intact (WT) and mutated target motifs. The sequences of the target motifs are indicated by a black line, and the PAMs are underlined with a red hatch. The arrows indicate the fragments deleted from the WT sequence (or inserted into) and the corresponding ligation points in mutant alleles.

	Plgn3Glc	Cy3Glc	Pn3Glc	Dp3Glc	Pt3Glc	Mv3Glc
m/z (total/aglycone)	433/271	449/287	463/301	465/303	479/317	493/331
aromatic ring B	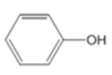	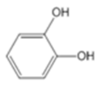	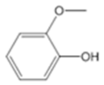	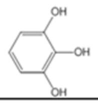	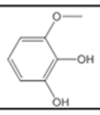	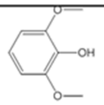

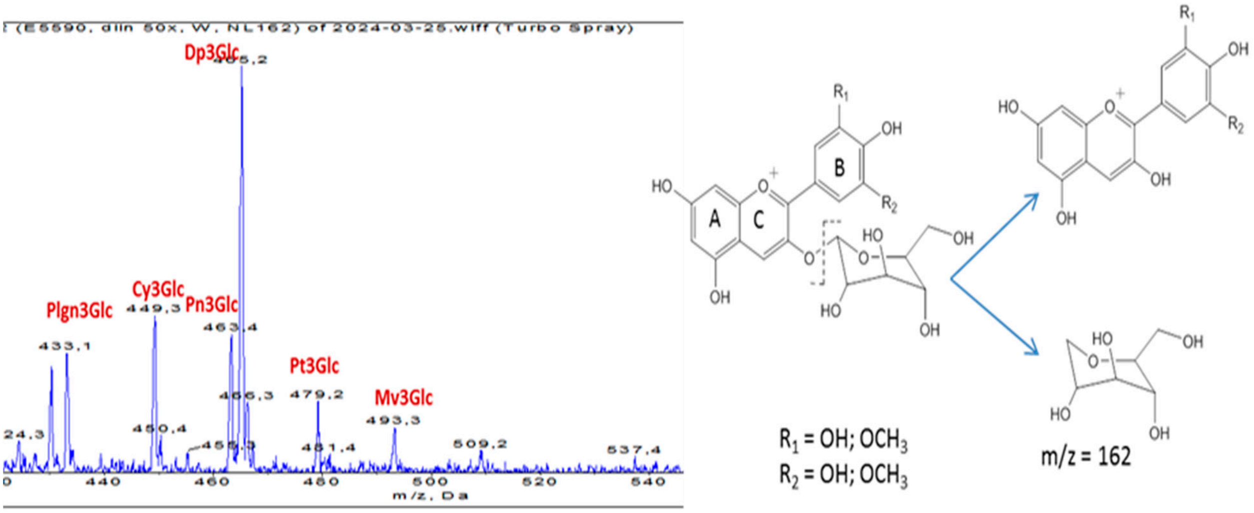

Figure S4. Mass spectrum of a total barley extract sample bAley (~20 µg/mL) recorded in the neutral fragment loss scanning mode m/z = 162. Direct input.

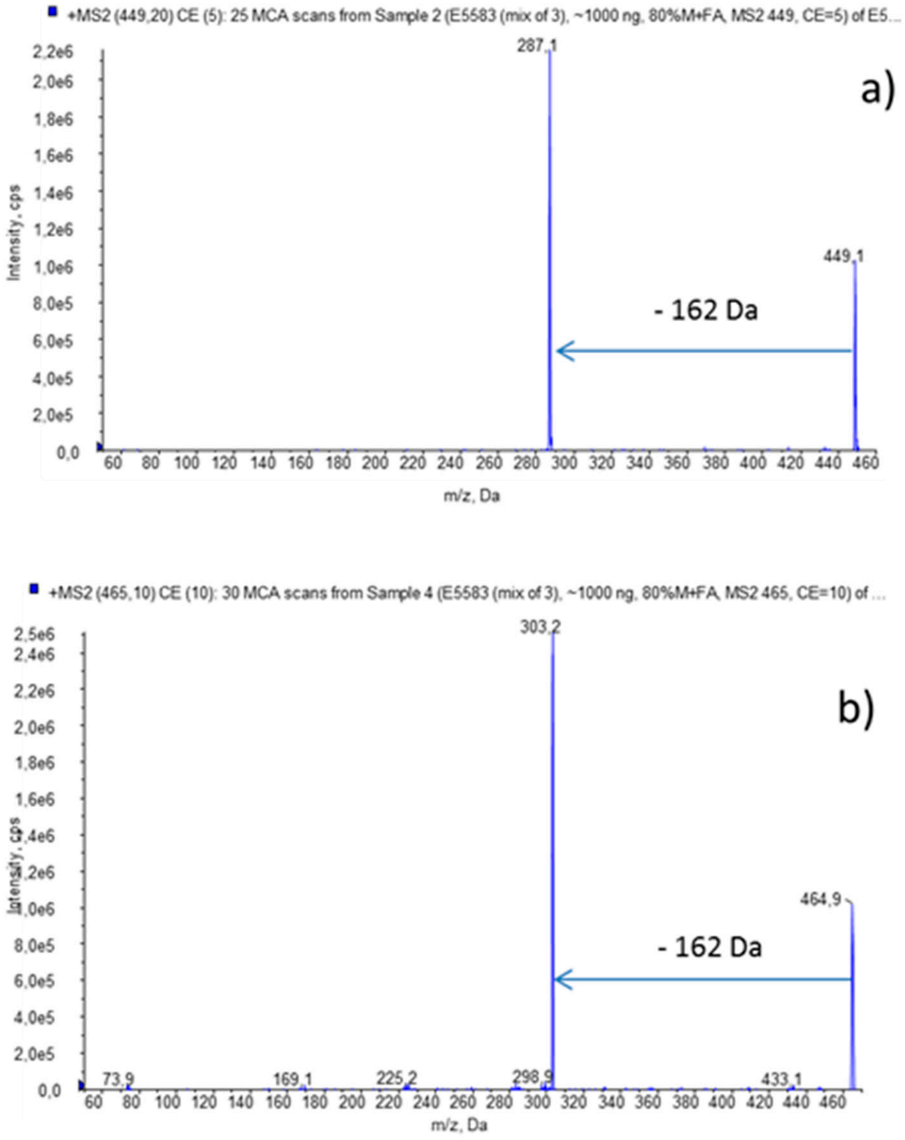

Figure S5. Mass spectra of the Cy3Glc (a) and Dp3Glc (b) in barley grain extracts.

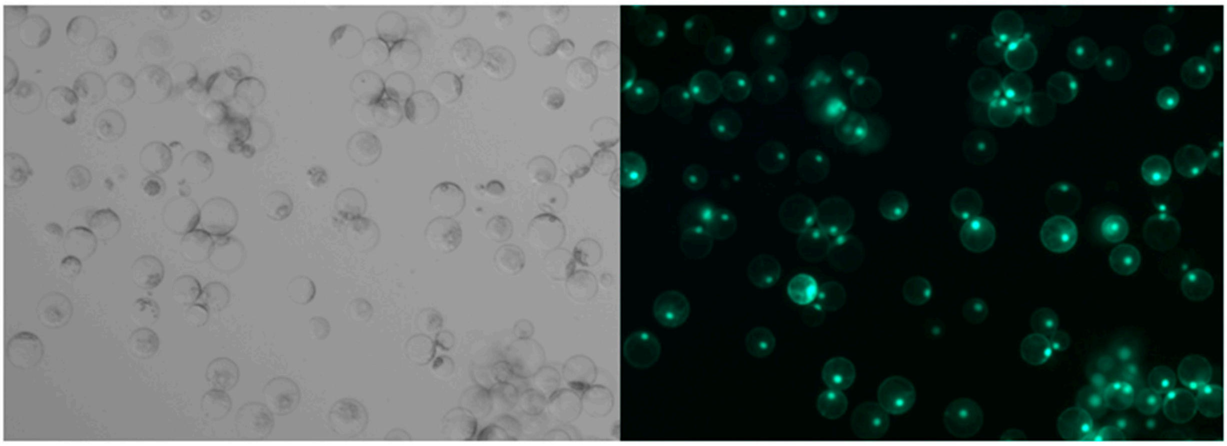

Figure S6. Identification of transformed protoplasts via GFP expression; bright field (left) and green filter (right). For *gfp* gene nuclear localization signal (NLS) derived from simian virus 40 (SV40) T antigen was installed as genetic fusion to direct the intracellular GFP protein to the nucleus of cells.

Supplemental methods

Method S1. High-performance liquid chromatography with diode array.

The extracts were analyzed by means of a liquid chromatograph (Agilent 1100-system, Agilent Technologies) equipped with a diode array detector. Separation was implemented on an Eclipse SB-C18 (4.6 × 150 mm, 5 µm) column in a binary solvent system consisting of 0.1% CF3COOH in water and methanol. The gradient of methanol was linear from 2% to 98% for 40 min, and the high-performance liquid chromatography (HPLC) runs were monitored at 520 nm for anthocyanins. Identification and quantification of anthocyanins were based on comparisons of the UV data with HPLC retention times of standards of cyanidin-3-glucoside, delphinidin-3-glucoside, and malvidin-3-glucoside (Merck, Germany) as well as mass spectrometry (MS) detection of separated compounds.

Method S2. High-performance liquid chromatography with mass-selective detector.

Analysis of the qualitative composition of anthocyanidins in a extract of bAley grain was carried out using HPLC/MS in MRM mode (multiple reaction monitoring, MRM) by scanning the fragmentation transitions of major molecular ions in the mode of neutral mass loss 162, found during the analysis of the sample in direct input mode. A Shimadzu LC-20AD Prominence chromatograph with a thermostatted autosampler SIL-20AC was used. The mobile phase consisted of eluent A - water with 0.1% HCOOH, eluent B - methanol with 0.1% HCOOH; flow rate 250 µl/min. A reversed-phase column was used with the following characteristics: ProntoSIL C18AQ, 2.1×75 mm (Econova, Novosibirsk), sorbent particle diameter 5 µm. HPLC analysis conditions (gradient elution) were: 0 min - 1% B, 0.5 min - 1% B, 3 min - 95%B, 7 min - 95% B. Then the column was equilibrated for the next analysis. A SCIEX 3200 QTRAP mass spectrometer (SCIEX, USA) with the following operating parameters was used as the detector: curtain gas pressure CUR=40 psi; ion source voltage IS=5500 V; dryer gas temperature TEM=200 C; spray gas pressure GS1=30 psi; desiccant gas pressure GS2=30 psi; collision cell gas pressure CAD=Low; cell entrance potential EP=10.0 V. Data were acquired in Analyst 1.6.3 (SCIEX) software supplied with the instrument. Chromatograms were processed in MultiQuant 2.1 program (SCIEX) in automatic integration mode
